# Frequency-Diverse Computational Direction of Arrival Estimation Technique

**DOI:** 10.1038/s41598-019-53363-3

**Published:** 2019-11-13

**Authors:** Okan Yurduseven, Muhammad Ali Babar Abbasi, Thomas Fromenteze, Vincent Fusco

**Affiliations:** 1 Centre for Wireless Innovation (CWI), Institute of Electronics, Communications and Information Technology (ECIT), School of Electronics, Electrical Engineering and Computer Science (EEECS), Queen’s University Belfast, Belfast, BT3 9DT UK; 2 XLIM Research Institute, University of Limoges, Limoges, 87060 France

**Keywords:** Electrical and electronic engineering, Engineering

## Abstract

We present a frequency-diverse based direction of arrival (DoA) estimation technique for millimetre-wave (mmW) 5G channel sounding. Frequency-diversity enables the creation of spatially incoherent radiation masks to encode the plane-wave signals incident on the radar aperture using a single antenna. Leveraging the frequency-diversity concept, spatial information of the plane-wave projections on the radar aperture is retrieved, resulting in high-fidelity DoA estimations by means of a simple Fourier transform operation applied to the retrieved plane-wave projection patterns. It is demonstrated that using the frequency-diversity concept, DoA estimation can be achieved through a simple frequency sweep, compressing the incoming plane-waves into a single channel through the transfer function of the radar aperture. This results in a significant simplification in the system hardware, requiring only a single antenna to achieve DoA estimation. It is also shown that the proposed technique can simultaneously detect the DoA information for multiple sources with a diffraction limited resolution.

## Introduction

Direction of Arrival (DoA) estimation plays a crucial role in a variety of applications, from navigation to channel estimation in wireless communications, including path delay characterization and localization. Using millimetre-wave (mmW) radiation for direction finding brings several advantages, such as all-weather operation, non-ionizing radiation, seeing through most optically opaque materials and mature component technology available at mmW frequencies. Despite these advantages, conventional DoA acquisition suffers from a number of challenges. Most DoA estimation techniques rely on an array-based hardware architecture, such as Bartlett algorithm^1–3^, Capon algorithm^4–7^, MUSIC algorithm^8–12^ and ESPRIT algorithm^13–17^. Because these techniques require beam synthesis on the receiver side, the channel information is collected by means of synthesizing a composite aperture either mechanically, such as in synthetic aperture radar (SAR)^17–19^, or electronically, such as in phased arrays^20–24^. Mechanical scanning is not desirable in that the data acquisition time can be large, posing a significant challenge for real-time operation. All-electronic operation can be achieved using phased arrays, however, beam synthesis using phased arrays requires that each antenna within the composite aperture is equipped with a phase-control circuit, or a phase-shifter, and preferably a power amplifier (to compensate for the insertion losses of the phase shifters). As a result, using phased arrays, all-electronic operation can require a large number of phase-shifting circuits and power amplifiers, significantly increasing the complexity and power-consumption of such apertures.

The concept of frequency-diversity has recently gained significant traction in computational imaging^25–36^, where the scene information can be encoded onto a set of measurement modes that exhibit quasi-randomness across the operating frequency band. In other words, the scene information is sampled on quasi-random bases at each frequency by means of stepping through a number of frequency points across the operating frequency band. Leveraging these engineered, intelligent modes eliminate the need for beam-synthesis and requires a simple frequency-sweep to capture the scene information. A three-dimensional (3D) microwave image of the scene (in this case the DoA of incoming signals) can then be reconstructed by interacting the measurements of the scene with the transfer function of the frequency-diverse imaging system using computational reconstruction techniques, such as matched-filtering and least-squares algorithms^25^.

Frequency-diversity in radar is a computational technique, relying on the generation of spatially-incoherent radiation patterns to retrieve an objective function. The application of frequency-diversity to imaging problems has proven to be promising to significantly simplify the physical constraints associated with the hardware layer^25–36^. Although sharing some similarities, DoA estimation^37^ differs from the imaging problem^38^ at a fundamental level. The concept of frequency-diverse imaging has mainly focused on the near-field operation, suggesting that the imaged scene is located within the near-field of the radar aperture. Moreover, frequency-diverse imaging relies on backscattered radar measurements. DoA estimation, on the other hand, is a far-field problem and there is no a-priori information available regarding the location of the source, such as a region of interest (RoI) surrounding an imaged object. This is due to the fact that in DoA estimation, the sources are placed in the far-field and it is only possible to detect their incident angles in 3D space, which is known as the DoA problem.

In this paper, we demonstrate the application of frequency-diversity to the DoA estimation problem for mmW 5G channel sounding. The studied scenario in this paper consists of a frequency-diverse aperture with only a single channel data acquisition hardware. In this paper, we show that the proposed DoA estimation technique can retrieve the channel information in a reliable manner and is robust to system noise. It is also shown that the proposed technique is only susceptible to the incident angle (*θ*, *φ*) parameters of the source and operates independently from possible variations in phase references when multiple sources are present. The outline of this paper is as follows: In the methods section, we explain the concept of frequency-diversity and its application to the DoA estimation problem. In the results and discussion section, we study several DoA estimation scenarios that include single source and multiple source examples. We also investigate the effect of several parameters on the DoA estimation performance of the proposed technique, such as system noise, quality-factor (Q-factor) of the frequency-diverse aperture and phase reference variations between multiple sources. The angular resolution limit is also studied to confirm the diffraction limited resolution characteristics of the proposed DoA estimation technique. Finally, the conclusions section provides the concluding remarks.

## Methods

### Forward model for DoA estimation

**Figure 1:**
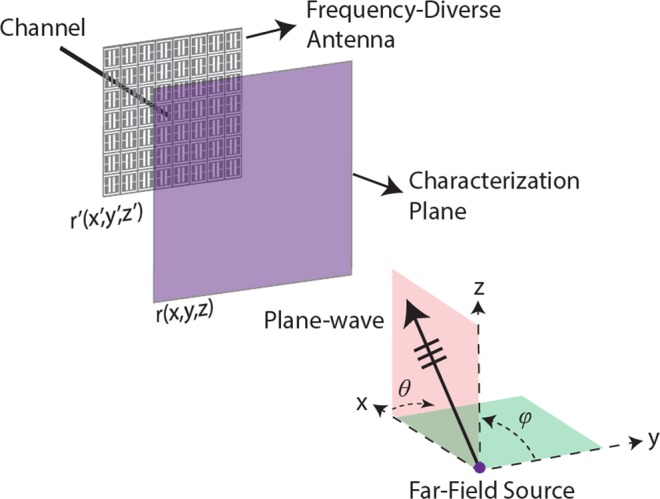
DoA estimation using the frequency-diversity technique. The far-field source generates a plane-wave illumination incident on the aperture at (*θ*, *φ)*. Not drawn to scale.

DoA estimation using frequency-diversity requires that a forward model is established to compress the incoming plane-waves through the transfer function of the frequency-diverse antenna. Figure 1 depicts a frequency-diverse radar aperture illuminated by a plane-wave incident at (*θ*, *φ*).

The frequency-diverse aperture shown in Fig. 1 exhibits a single channel and is designed to operate between 27–29 GHz, centred around the 28 GHz 5 G frequency band. The size of the aperture is 30 cm × 30 cm, corresponding to an electrical size 28*λ*_0_ × 28*λ*_0_, where *λ*_0_ is the free-space wavelength at 28 GHz. Assuming an exponential decay, the time domain impulse response of the frequency-diverse antenna is proportional to the antenna Q-factor by $h(t)\propto {e}^{-2t/\tau }$, where $Q={\omega }_{0}\tau /2$ and ${\omega }_{0}$ is the resonance frequency of each mode within the operating frequency band. Therefore, the width of the aperture impulse response is governed by the Q-factor of the aperture. Increasing the Q-factor of the antenna also increases the width of the impulse response, which in return, reduces the correlation between the measurement modes^26^. It is therefore desirable that the Q-factor of the antenna is selected to be large.

This paper aims to develop a proof-of-concept for the application of the frequency-diversity technique to far-field DoA estimation problems. Particularly, we focus here on the 5G channel sounding problem as the application of interest and note that the presented technique holds and can also be applied to other classical radar problems, such as tracking and remote sensing to name a few. This proof-of-concept study relies on analytical modelling of the radar aperture and numerical simulations of the frequency-diversity technique for various DoA estimation scenarios. The developed analytical framework and numerical simulations enable us to study various system parameters independent of experimental constraints, ensuring systematic studies to be carried out to prove the frequency-diverse DoA concept. For example, using the developed framework, we have a freedom to choose an arbitrarily large Q-factor, analyse the variation of the Q-factor, investigate the effect of system noise and study a varying number of far-field sources incident on the radar aperture for DoA estimation.

Ultimately, the number of useful measurement modes produced by a frequency-diverse aperture can be given as *QB*/*f*_*c*_^30^, where *B* is the operational bandwidth and *f*_*c*_ is the centre frequency. For this study *B* = 29 GHz–27 GHz = 2 GHz while *f*_*c*_ = 28 GHz and we choose the Q-factor to be *Q* = 12000. Following the *QB*/f_c_ mode upper-bound limit derived in^30^, this selection suggests that the number of useful measurement modes that the system depicted in Fig. 1 can produce is around *M*_*max*_ = 860.

Following the definition of the frequency-diverse radar aperture, we choose a plane located in the near-field of the aperture as depicted in Fig. 1. Throughout this paper, this plane will be referred to as the *characterization plane*. Defining the characterization plane is essential to establish the forward model in that the modes radiated by the frequency-diverse radar aperture are calculated on this plane and this characterization is vital to know the transfer function of the radar aperture to an impulse excitation corresponding to a frequency-bandwidth of 27–29 GHz. The transfer function of the frequency-diverse aperture on the characterization plane can be obtained analytically or experimentally. Experimental characterization is conventionally achieved using a near-field scanner measuring the radiated field data on the characterization plane^26^. In this work, we follow the analytical approach. The frequency-diverse antenna in Fig. 1 is a metasurface aperture, where the aperture surface is loaded with an array of meta-elements. At a subwavelength limit, the radiation of each meta-element can be modelled as a magnetic dipole, $m(\omega ,r^{\prime} )=\alpha (\omega ,{\omega }_{0},r^{\prime} )H(\omega ,r^{\prime} )$, where *α* represents the element polarizability, *H* is the magnetic field that excites the complementary meta-atoms, *r*′ is the position index on the metasurface aperture, *ω* is the frequency, and *ω*_0_ is the resonance frequency of the meta-elements that are randomly distributed spatially across the metasurface aperture. The frequency-diverse metasurface aperture modelled in this work has a planar architecture and is fed through a single channel located at the aperture centre. The element polarizability follows a Lorentzian distribution, $\alpha =\frac{{\omega }^{2}}{{\omega }_{0}^{2}-{\omega }^{2}+j\varGamma \omega }$, where Γ is the damping factor^36^. The launched magnetic field by the centre feed can be calculated as $H={H}_{0}^{1}k(r^{\prime} )$, where ${H}_{0}^{1}$ is the Hankel function (zeroth order and first kind) and *k* is the wavenumber within the antenna aperture^39,40^. Following the coordinate system definition in Fig. 1, the projection of the aperture radiated fields, *E(ω*, *r)*, on the characterization plane can be calculated as follows:

1 $$E(\omega ,r)={\int }_{r^{\prime} }m(r^{\prime} )G(\omega ,r,r^{\prime} )dr^{\prime} $$

In Eq. (1), *r* denotes the coordinates of the characterization plane, *k*_0_ is the wavenumber in free space and the Green’s function is given by $G={e}^{-j{k}_{0}|r-r^{\prime} |}$. The aperture radiated field projected onto the characterization plane, *E(ω*, *r)*, is the transfer function of the frequency-diverse antenna.

The next step in the DoA estimation problem is to calculate the projection of the incident far-field source on the characterization plane. Using the same coordinate system in Fig. 1, the far-field projection can be calculated as follows:

2 $$P={e}^{-j{k}_{0}(y\sin \theta \cos \phi +z\sin \theta \sin \phi )}$$

In Eq. (2), (*θ*, *φ*) denote the incident angles of the source to be retrieved through the proposed DoA estimation technique. In Eq. (1) and Eq. (2), we note that the DoA estimation fidelity is governed mainly by the phase information and hence the amplitude dependence is dropped. The final step in establishing the forward model is the compression of the plane-wave projection pattern on the characterization plane into the single channel of the frequency-diverse aperture. This is achieved through the transfer function of the aperture on the characterization plane as follows:

3 $$g(\omega )={\int }_{r}E(r,\omega )P(r)dr+n$$

In Eq. (3), *g* is the compressed signal and will be referred to as the *measured signal* throughout this paper and *n* denotes the measurement noise.

### DoA estimation using frequency-diversity

The DoA estimation problem is inherently interested in retrieving an estimate of the plane-wave source projection pattern on the characterization plane. Therefore, the source projection pattern on the characterization plane is the objective function for the presented DoA estimation problem. Discretizing Eq. (3) and using the transfer function of the frequency-diverse aperture, an estimate of the source projection pattern on the characterization plane can be retrieved from the compressed measurement as follows:

4 $${P}_{est}={E}^{\dagger }g$$

In Eq. (4), a phase conjugated transfer function of the frequency-diverse aperture is applied to the compressed measurement to retrieve an estimate of the objective function, *P*_est_, which is known as matched-filtering. In this work, we make use of a least-squares reconstruction algorithm to minimize the error residue in the estimation of the objective function in an iterative manner as follows:

5 $${P}_{est+1}=\text{arg}\,\min \,{\Vert g-E{P}_{est}\Vert }_{2}^{2}$$

Following the retrieval of an estimate of the source projection pattern on the characterization plane, the DoA estimation can be achieved by means of a simple Fourier transform operation applied to the estimated source projection pattern, $\Im ({P}_{est})$. Using a peak-finding algorithm on the estimated DoA pattern, the incident angles *(θ*, *φ*) can be retrieved.

## Results and Discussion

**Figure 2:**
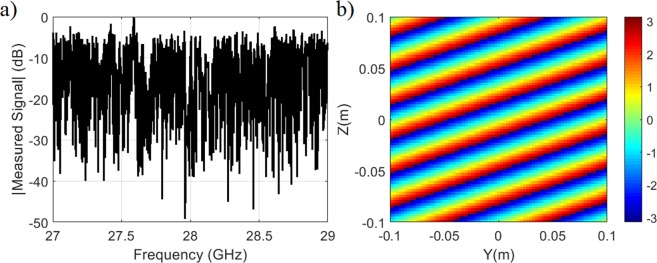
(**a**) Frequency-dependent compressed signal at the antenna channel (**b**) far-field projection pattern (original) on the characterization plane (phase in radians).

Following the frequency-diverse system depicted in Fig. 1, we begin with the DoA estimation scenario consisting of a single source placed in the far-field of the radar aperture. For this study, the size of the frequency-diverse aperture is *D* = 0.3 m while the incident angle of the source is selected to be (*θ* = 20°, *φ* = −20°). The characterization plane is positioned at *d* = 0.2 m from the aperture of the frequency-diverse antenna. The number of frequency points is selected to be in accordance with the upper-bound limit on the number of useful measurement modes calculated earlier, *M* = 860. The compressed signal measured at the channel of the antenna is shown in Fig. 2(a) while the original (or ground truth) plane-wave projection on the characterization plane is shown in Fig. 2(b).

**Figure 3:**
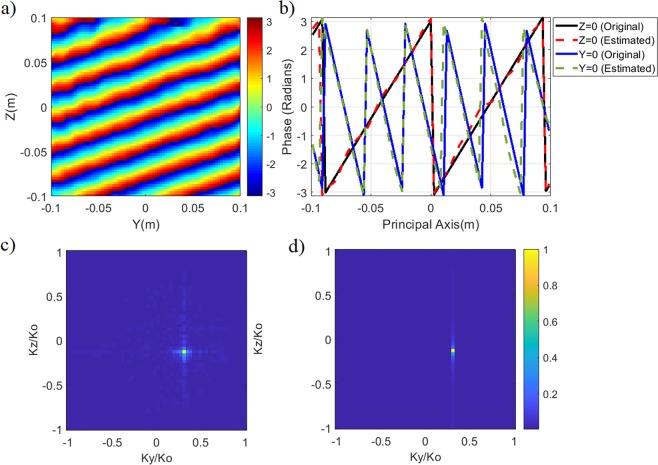
DoA estimation (**a**) retrieved source projection on the characterization plane (phase in radians) (**b**) phase difference between original and retrieved source projection patterns across the principal axes of the characterization plane (**c**) reconstructed DoA pattern (**d**) actual DoA pattern (original). For DoA patterns, normalized magnitude is shown.

The retrieved source projection pattern on the characterization plane, *P*_est_, is shown in Fig. 3(a). In Fig. 3(b), the phase difference between the original and retrieved source projection patterns is shown along two principal axes, *y* = 0 and *z* = 0. From Fig. 3 it is evident that the retrieved source projection pattern is in good agreement with the original pattern with the average phase error being calculated to be smaller than π/18 radians. Taking the Fourier transform of the original and retrieved source projections, we obtain the DoA patterns shown in Fig. 3(c,d).

Running a peak searching algorithm in Fig. 3(a), the normalized wave-vectors are found to be ${K}_{y}/{K}_{0}=$ $\sin \,\theta \,\cos \,\phi =0.32$ and ${K}_{z}/{K}_{0}=\,\sin \,\theta \,\sin \,\phi =-\,0.11$, resulting in *θ*_est = _19.8°, *φ*_est_ = −19.1°, which is in good agreement with the ground truth, (*θ* = 20°, *φ* = −20°).

### Effect of noise

**Figure 4:**
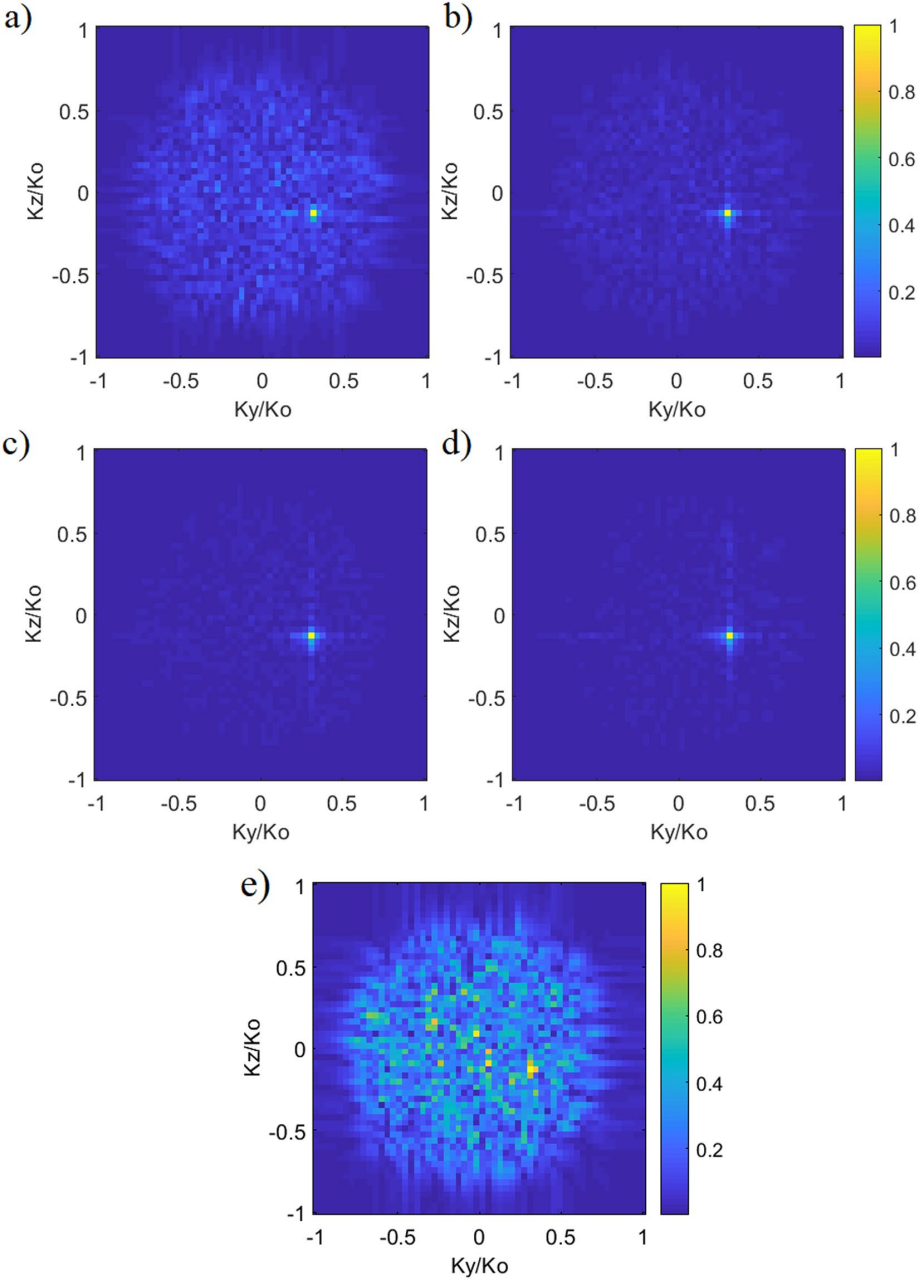
Reconstructed DoA estimations (normalized magnitude) as a function of varying noise levels (**a**) SNR = 0 dB (**b**) SNR = 5 dB (**c**) SNR = 10 dB (**d**) SNR = 20 dB. SNR can have a substantial effect on estimation performance. DoA pattern with SNR = −20 dB is shown in (**e**).

The DoA estimation study shown in Fig. 3 does not include noise in the compressed channel, suggesting that the signal to noise (SNR) ratio for the studied scenario is infinite. In reality, the DoA estimation problem will exhibit channel induced measurement noise, resulting in a finite SNR level. To analyse the performance of the proposed DoA estimation technique, we add a Gaussian white noise to the compressed signal and perform a parametric SNR sweep to observe the DoA response of the frequency-diverse radar. The estimated DoA patterns are demonstrated as a function of varying SNR in Fig. 4. For this study, the number of measurement modes is *M* = 860.

Analysing Fig. 4, it can be concluded that the retrieved plane-wave reconstructions appear to be robust to system noise above SNR = 5 dB level. As an extreme example, reducing the SNR level to −20 dB, it is evident that the DoA estimation fails. Analysing the retrieved DoA estimation patterns as a function of SNR in Fig. 4 reveals another important outcome. In the presented analytical model in Eq. (3), the measured signal, *g*, and the far-field projection pattern, *P*, are correlated by the transfer function of the frequency-diverse antenna, *E*. A model error caused by various discrepancies in this correlation in Eq. (3) would also appear as a noise term added to the measurements. Hence, Eq. (3) with the added noise term is a good representation not only for systematically analysing the SNR ratio for the channel but also observing the effect of such discrepancies added to the forward model because the noise has a complex form. The noise study carried out in Fig. 4 shows that, despite the large Q-factor of the antenna, the system is robust to such variations.

### DoA estimation of multiple sources

**Figure 5:**
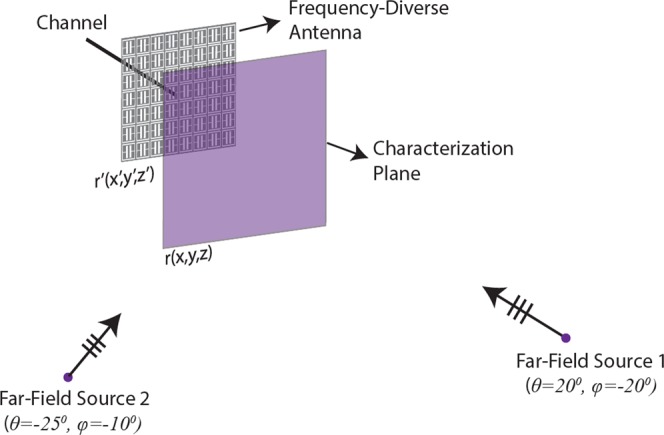
DoA estimation problem with multiple far-field sources. Not drawn to scale.

Although the DoA estimation demonstration for a single far-field source is important to explain how frequency-diversity can be adopted to the DoA problem, a practical DoA scenario requires the direction estimation for multiple plane-wave sources located arbitrarily in a 3D space. To achieve this, we define two sources positioned in the far-field of the frequency-diverse aperture, illuminating the aperture at (*θ*_1_ = 20°, *φ*_1_ = −20°) and (*θ*_2_ = −25°, *φ*_2_ = −10°) incident angles as depicted in Fig. 5.

For the multiple source problem, the source projection patterns on the characterization plane are superposed and compressed by the transfer function of the frequency-diverse aperture as follows:

6 $${P}_{T}={P}_{1}+{P}_{2}={e}^{-j{k}_{0}(y\sin {\theta }_{1}\cos {\phi }_{1}+z\sin {\theta }_{1}\sin {\phi }_{1})}+{e}^{-j{k}_{0}(y\sin {\theta }_{2}\cos {\phi }_{2}+z\sin {\theta }_{2}\sin {\phi }_{2})}$$

7 $$g(\omega )={\int }_{r}E(r,\omega ){P}_{T}(r)dr$$

**Figure 6:**
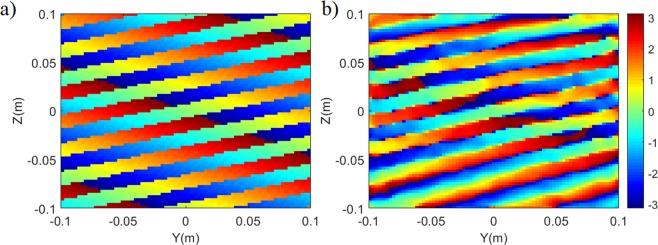
Source projection patterns in the characterisation plane (**a**) original (**b**) reconstructed. For both patterns phase is shown in radians.

In Eq. (6), *P*_1_ is the projection of the first source on the characterization plane while *P*_2_ refers to the projection of the second source on the characterization plane. The original and retrieved source projection patterns in the characterization plane are shown in Fig. 6.

**Figure 7:**
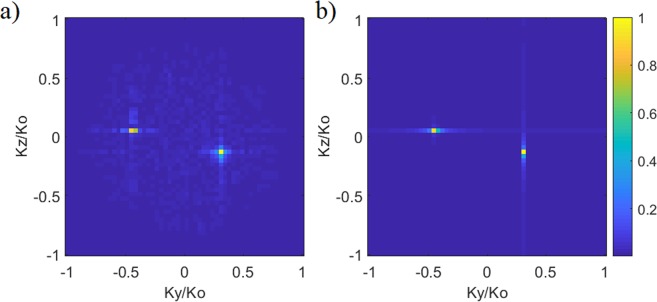
Reconstructed DoA estimation pattern for the multi-source scenario (**a**) reconstructed DoA pattern (**b**) actual DoA pattern (original). Normalized magnitude is shown.

Comparing the projection patterns in Fig. 6, the average phase error is calculated to be smaller than π/9 radians. Taking the Fourier transforms of the estimated and original projection patterns, the calculated DoA patterns are shown in Fig. 7.

From Fig. 7, the normalized wave-vectors are found to be ${K}_{y1}/{K}_{0}=\,\sin \,{\theta }_{1}\,\cos \,{\phi }_{1}=0.32$ and ${K}_{z1}/{K}_{0}=$ $\sin \,{\theta }_{1}\,\sin \,{\phi }_{1}=-\,0.11$ for source 1 and ${K}_{y2}/{K}_{0}=\,\sin \,{\theta }_{2}\,\cos \,{\phi }_{2}=-\,0.41$ and ${K}_{z2}/{K}_{0}=\,\sin \,{\theta }_{2}\,\sin \,{\phi }_{2}=0.065$ for source 2. From the estimated normalized wave-vector indices, the DoA estimation for sources 1 and 2 are calculated to be (*θ*_1est_ = 19.8°, *φ*_1est_ = −19.1°) and (*θ*_2est_ = −24.5°, *φ*_2est_ = −9.3°), respectively. The estimated results exhibit good agreement with the actual DoA data, (*θ*_1_ = 20°, *φ*_1_ = −20°) and (*θ*_2_ = −25°, *φ*_2_ = −10°). The result presented in Fig. 7 is a testament to the fact that the proposed frequency-diverse DoA technique can achieve direction finding for multiple sources. It is also important to note that the plane-wave sources for the scenario studied in Fig. 7 are asymmetrically distributed in space. This is to test that the proposed frequency-diverse DoA technique does not rely on symmetrical source distribution and the approach can retrieve the incident angles of sources irrespective of their positioning.

### Effect of Q-factor

**Figure 8:**
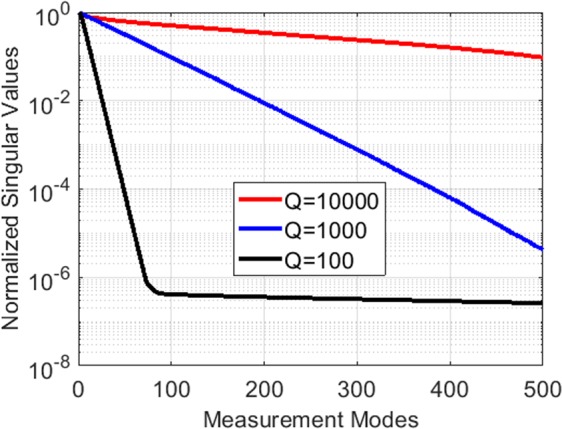
Singular values of the frequency-diverse antenna fields on the characterization plane as a function of Q-factor (**a**) *Q* = 100 (**b**) *Q* = 1000 (**c**) *Q* = 10000.

The Q-factor of the frequency-diverse aperture governs the impulse response of the aperture. The correlation between the frequency-diverse radiated field patterns on the characterization plane is governed by the transfer function of the antenna. In order to analyse the effect of the Q-factor on the encoding of the objective function and determine the quality of mode orthogonality, we perform a singular value decomposition (SVD) analysis^26^. To this end, the antenna radiated field data on the characterization plane is fed to an SVD algorithm analysing the distribution of the singular values as a function of varying Q-factors. The calculated singular value patterns are shown in Fig. 8. The results validate that increasing the Q-factor of the antenna improves the conditioning of the DoA estimation problem, and hence it is desirable to select as large a Q-factor as possible.

**Figure 9:**
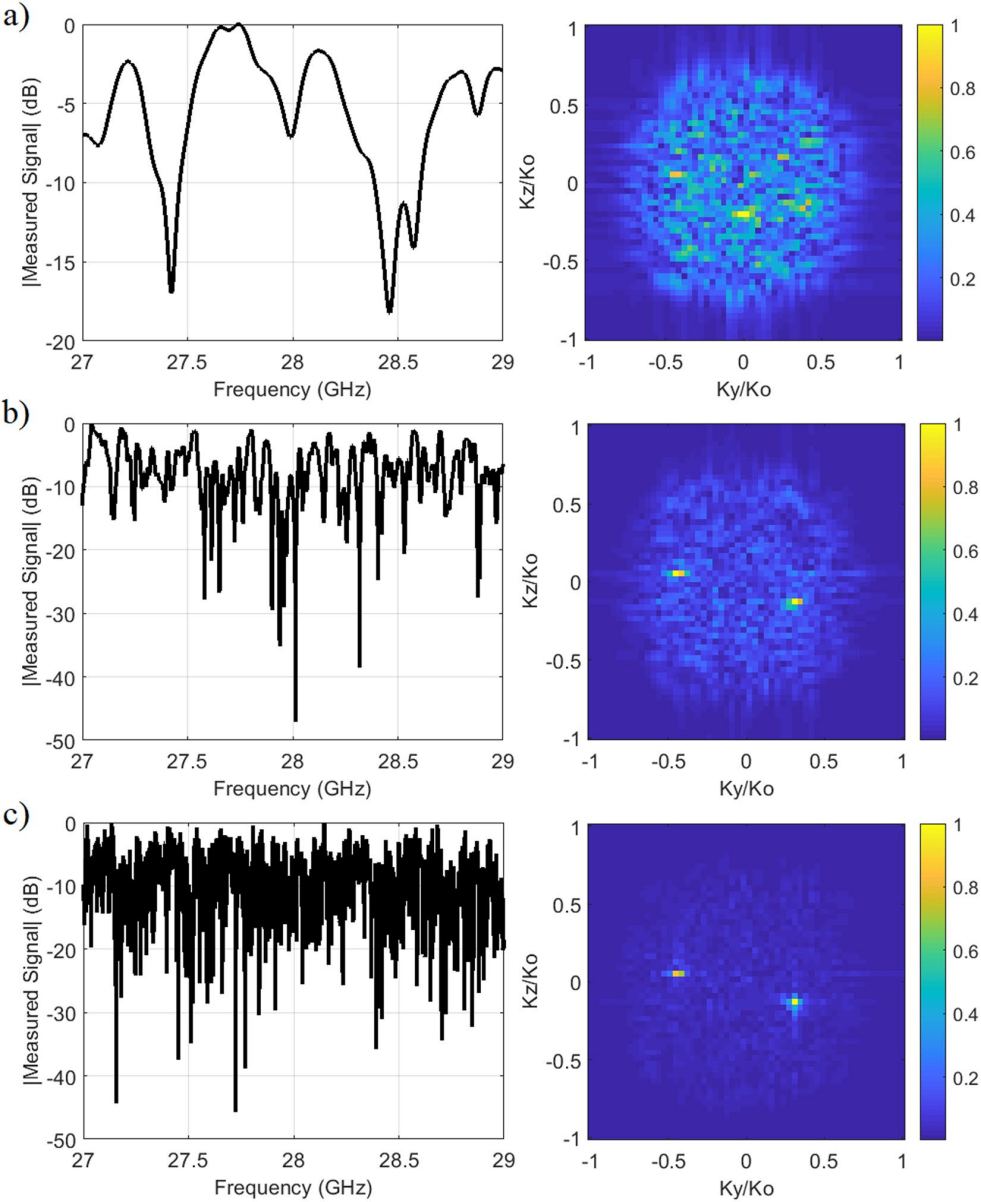
Measured signal at compressed channel of the frequency-diverse antenna (left) and the retrieved DoA pattern (right) as a function of Q-factor (**a**) *Q* = 100 (**b**) *Q* = 1000 (**c**) *Q* = 10000. The number of modes with minimized information redundancy can be calculated as 8, 72 and 715, respectively^30^. For DoA patterns, normalized magnitude is shown.

To confirm this observation, in Fig. 9, we study the multi-source DoA estimation problem as a function of varying the Q-factor. Analysing the retrieved DoA patterns, it is evident that increasing the Q-factor of the antenna results in a superior DoA estimation. Comparing the measured signal patterns, it can be seen that the sharpness of the resonances in the compressed measured signal data is directly proportional to the Q-factor, with the frequency-diverse aperture exhibiting narrower resonances as the Q-factor of the aperture is increased.

Because the presented work leverages a small fractional bandwidth (7.1%), it is important that the Q-factor of the antenna is selected to be large to decorrelate the radiated field patterns at adjacent frequency points within the operating bandwidth. From a practical perspective, increasing the Q-factor of a frequency-diverse antenna can be achieved by reducing the losses, particularly the conduction and dielectric losses^32^. Although this work aims to present a proof-of-concept validation of the frequency-diverse DoA estimation technique, we note that, for a practical system, an air-filled cavity-backed metasurface antenna^33^ can be considered as an ideal candidate to achieve this requirement. At mmW frequencies, an important factor that affects the overall system cost implications would be the requirement to achieve surface roughness values on the order of or smaller than *λ*/20, which might easily be realized by polishing the internal walls of the antenna.

It should be mentioned that the peak-finding algorithm applied to the retrieved DoA estimation patterns consists simply of finding the index values of maximum magnitude points corresponding to the arrival angle information. This is a simple and linear process, and is adequate for most of the scenarios studied in this paper where the Q-factor and the SNR level are selected to be sufficient to ensure high fidelity DoA pattern retrievals. However, for more complex scenarios, such as the scenario studied in Fig. 9(a), more sophisticated peak finding algorithms might be needed.

### Effect of phase reference between sources

**Figure 10:**
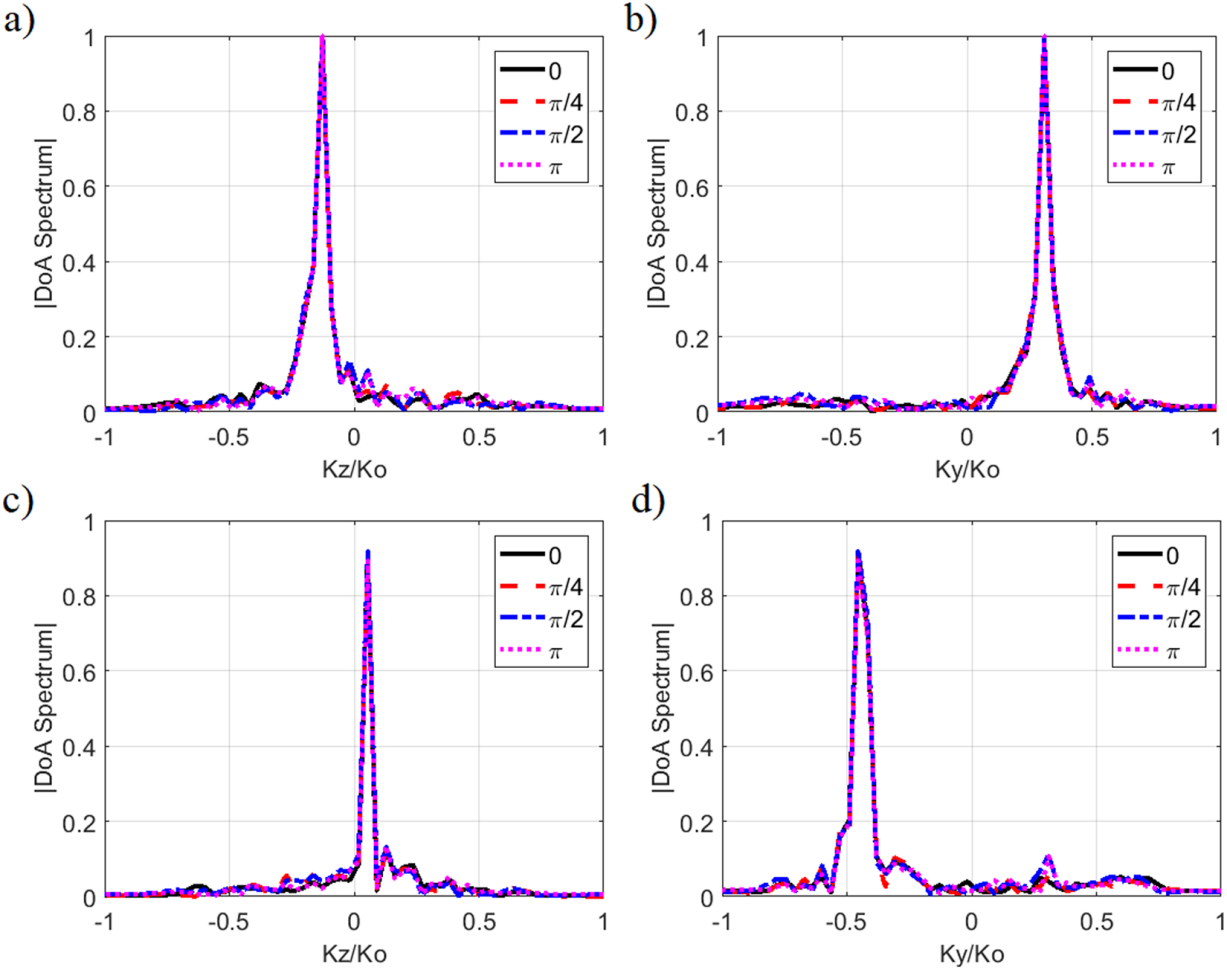
Principal projections of the reconstructed normalized DoA patterns as a function of varying phase reference offset added to source 1. Source 1: (**a**) DoA spectrum along normalized *k*_*z*_ axis (**b**) DoA spectrum along normalized *k*_*y*_ axis. Source 2: (**c**) DoA spectrum along normalized *k*_*z*_ axis (**d**) DoA spectrum along normalized *k*_*y*_ axis. Phase reference offset is varied from 0 radians to π radians.

An important parameter to investigate for the presented DoA estimation technique is the effect of arbitrary phase references when multiple sources are present. In a practical wireless environment, there is no requirement for different sources to share the same phase reference and, therefore, it is vital that the presented DoA estimation technique works independently of the phase reference variations between the multiple sources. To study the effect of varying the phase reference, we consider the two-source scenario, (*θ*_1_ = 20°, *φ*_1_ = −20°) and (*θ*_2_ = −25°, *φ*_2_ = −10°), and introduce an arbitrarily selected phase difference between the two sources by shifting the reference phase of source 1 within the range of 0–π radians. The estimated DoA pattern projections along the normalized *k*_*y*_ and *k*_*z*_ axes are shown in Fig. 10.

Analysing Fig. 10, it is evident that the presented frequency-diverse DoA estimation technique is sensitive to the incident (*θ*, *φ*) angles and operate independently from phase reference variations present between the multiple sources.

### Angular resolution limit

**Figure 11:**
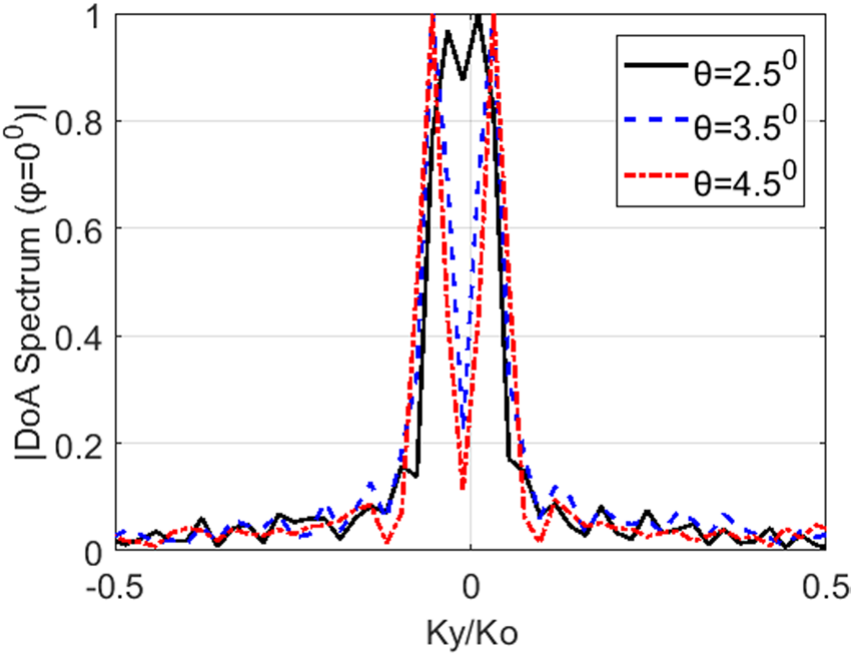
DoA patterns across the *φ* = 0° plane as a function of *θ* separation in between the incident far-field sources.

The far-field diffraction limited resolution for a square aperture can be given as *δ* = *λ*_0_/*D*, where *λ*_0_ is the free-space wavelength and *D* is the aperture size. Accordingly, for the studied DoA estimation problem, the angular resolution is governed by the frequency band of operation and the size of the radar aperture. The computational nature of the presented frequency-diverse DoA estimation technique enables the DoA estimation to be achieved using a single, monostatic radar aperture, bringing a significant simplification on the physical hardware layer. However, it is also important to demonstrate that this hardware simplification is not achieved at the expense of the resolution characteristics of the DoA estimation system. To study the resolution limit of the frequency-diverse DoA estimation technique, we consider a parametric study of two plane-wave sources incident on the radar aperture that are separated by *θ* from each other. The retrieved DoA estimation patterns are observed as a function varying *θ* values and a cross section of the DoA estimation patterns is taken along the *φ* = 0° plane to analyse the interference between the corresponding source peaks in the DoA patterns. The DoA patterns across the *φ* = 0° plane are shown in Fig. 11.

In Fig. 11, the incident plane-waves are identified as separate sources starting from *θ* = 2.5° separation angle. Given the size of the frequency-diverse aperture, *D* = 0.3 m and considering the 28 GHz central frequency, the lower-bound theoretical limit can be calculated as 2.1°, which is in good agreement with the observed resolution limit.

### Full-wave simulations

**Figure 12:**
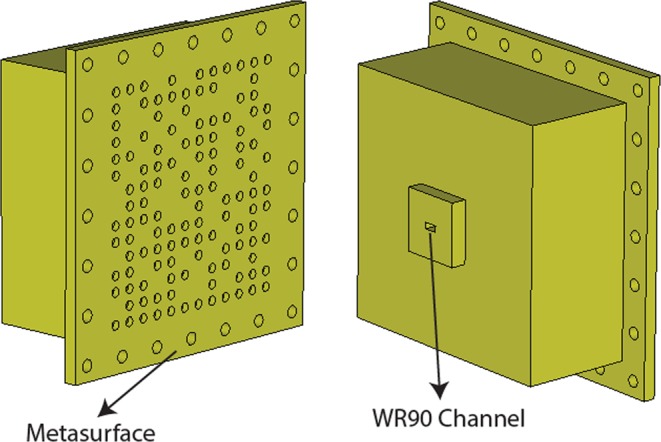
Designed cavity-backed metasurface frequency-diverse antenna for full-wave simulations.

In order to further validate the proposed frequency-diverse DoA estimation technique, in this section, we consider full-wave simulations. The simulations are conducted using a commercial EM simulation software, CST Microwave Studio, using a time-domain solver. The frequency-diverse antenna is built upon our previous work on computational polarimetric imaging^33^. The antenna consists of a metasurface layer with sub-wavelength, circular shaped irises excited using a cavity-backed feeding architecture. The cavity-backed feeding has a single waveguide port (WR90) as depicted in Fig. 12. It should be noted that, because of the computational complexity of the problem at hand, the frequency-band of operation is chosen to be 9–11 GHz within the X-band frequency regime (8–12 GHz). For the full-wave simulations, we consider two scenarios; case 1: on-axis incident (*θ* = 0°, *φ* = 0°) and case 2: oblique incident (*θ* = −15°, *φ* = 0°).

**Figure 13:**
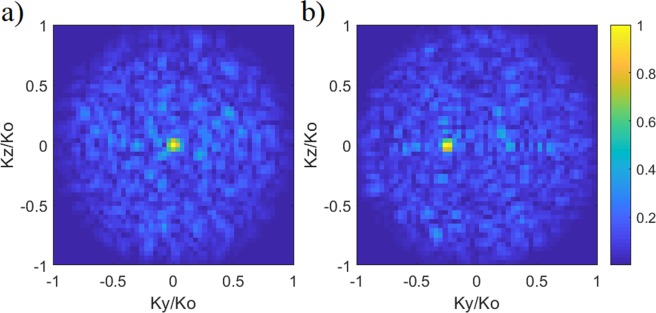
Retrieved DoA estimation patterns (normalized magnitude) (**b**) case 1: on-axis incident (*θ* = 0°, *φ* = 0°) (**b**) case 2: oblique incident (*θ* = −15°, *φ* = 0°).

The retrieved DoA estimation patterns for case 1 and case 2 are shown in Fig. 13.

Analysing Fig. 13, the DoA estimation is calculated to be (*θ*_est_ = 0°, *φ*_est_ = 0°) for case 1 and (*θ*_est_ = −15.2°, *φ*_est_ = 0°) for case 2. In addition to the good agreement between the actual and simulated DoA values, the presented results in Fig. 13 also demonstrate the scalability of the frequency-diverse DoA technique to different frequency bands.

## Conclusions

We have presented a frequency-diverse radar technique that relies on a set of compressive measurements to computationally retrieve the DoA estimation. The presented technique uses a single, monostatic radar aperture to capture the channel information and compress the incoming source patterns into a single channel. Therefore, it eliminates the need for aperture synthesis using an array of antennas and multiple channels to estimate DoA, significantly simplifying the hardware architecture. The presented technique has been proven for the 27–29 GHz frequency band centred around the 28 GHz frequency band and holds potential for mmW 5 G channel estimation. It has been shown that, by means of a simple frequency sweep, the incident angle of far-field sources can be estimated to a good accuracy. We have also further verified the presented technique using full-wave simulations at X-band frequencies. The observed results confirm that the frequency-diverse DoA estimation technique can readily be scaled to other frequencies with applications including smart antennas, mobile communications, navigation, sonar, radar tracking and radio astronomy.
